# Experiences and perceptions of conditional cash incentive provision and cessation among people with HIV for care engagement: A qualitative study

**DOI:** 10.21203/rs.3.rs-3905074/v1

**Published:** 2024-02-07

**Authors:** Julia Giordano, Jayne Lewis-Kulzer, Lina Montoya, Eliud Akama, Harriet Fridah Adhiambo, Everlyne Nyadieka, Sarah Iguna, Elizabeth A. Bukusi, Thomas Odeny, Carol S. Camlin, Harsha Thirumurthy, Maya Petersen, Elvin H. Geng

**Affiliations:** Washington University in St. Louis; University of California San Francisco; University of North Carolina at Chapel Hill; Kenya Medical Research Institute; Kenya Medical Research Institute; Kenya Medical Research Institute; Kenya Medical Research Institute; Kenya Medical Research Institute; Kenya Medical Research Institute; University of California San Francisco; University of Pennsylvania; University of California, Berkeley; Washington University in St. Louis

**Keywords:** HIV, care engagement, retention, conditional cash transfers, incentives, Africa

## Abstract

**Background:**

Consistent engagement in HIV treatment is needed for healthy outcomes, yet substantial loss-to-follow up persists, leading to increased morbidity, mortality and onward transmission risk. Although conditional cash transfers (CCTs) address structural barriers, recent findings suggest that incentive effects are time-limited, with cessation resulting in HIV care engagement deterioration. We explored incentive experiences, perceptions, and effects after cessation to investigate potential mechanisms of this observation.

**Methods:**

This qualitative study was nested within a larger trial, AdaPT-R (NCT02338739), focused on HIV care engagement in western Kenya. A subset of participants were purposively sampled from AdaPT-R participants: adults with HIV who had recently started ART, received CCTs for one year, completed one year of follow-up without missing a clinic visit, and were randomized to either continue or discontinue CCTs for one more year of follow-up. In-depth interviews were conducted by an experienced qualitative researcher using a semi-structed guide within a month of randomization. Interviews were conducted in the participants’ preferred language (Dholuo, Kiswahili, English). Data on patient characteristics, randomization dates, and clinic visit dates to determine care lapses were extracted from the AdaPT-R database. A codebook was developed deductively based on the guide and inductively refined based on initial transcripts. Transcripts were coded using Dedoose software, and thematic saturation was identified.

**Results:**

Of 38 participants, 15 (39%) continued receiving incentives, while 23 (61%) were discontinued from receiving incentives. Half were female (N = 19), median age was 30 years (range: 19–48), and about three-quarters were married or living with partners. Both groups expressed high intrinsic motivation to engage in care, prioritized clinic attendance regardless of CCTs and felt the incentives expanded their decision-making options. Despite high motivation, some participants reported that cessation of the CCTs affected their ability to access care, especially those with constrained financial situations. Participants also expressed concerns that incentives might foster dependency.

**Conclusions:**

This study helps us better understand the durability of financial incentives for HIV care engagement, including when incentives end. Together with the quantitative findings in the parent AdaPT-R study, these results support the idea that careful consideration be exercised when implementing incentives for sustainable engagement effects.

## Introduction

Rapid expansion of lifesaving antiretroviral therapy (ART) has resulted in substantial gains in longevity among people with HIV (PWH), yet sub-Saharan Africa (SSA) continues to lead in global HIV infections and suboptimal retention in HIV care (1–3). To achieve healthy outcomes and minimize morbidity, mortality and HIV transmission risk, PWH require consistent engagement in HIV care and treatment. However, in SSA, about one-third of PWH become lost-to-follow up after two years of ART initiation (1, 4, 5). Staying in care can be difficult given the number of challenges PWH face in SSA. Clinic-based barriers present challenges, where PWH may experience unfriendly healthcare workers or prolonged wait times (6, 7). Psychological barriers can also interfere with clinic attendance. For example, PWH may face stigma if they were to be seen by someone from the community while at the clinic or feel they may be judged negatively by providers during clinical encounters (8, 9). However, structural barriers are the most pronounced barriers to HIV care in low-resource settings, where patients are commonly faced with finding funds for transportation to the clinic and missing wages while away from work in a backdrop of widespread poverty and unemployment (2, 3, 8–10). Addressing structural challenges is critical for sustained patient engagement and viral suppression for healthy outcomes over time (1, 4, 11, 12).

Incentives in the form of conditional cash transfers (CCTs), or predictable payment vouchers is a method employed to address structural barriers and improve care attendance. The CCTs can be used to pay for specific things such as transportation costs (13, 14). The incentives aim to alleviate transportation costs to and from the clinic, with the added benefit that the extrinsic economic motivation ultimately may shift to an intrinsic motivation that persists even after the CCTs are discontinued (15). Evidence suggests that incentives can motivate people towards healthy behaviors. Incentives have been shown to help PWH remain in care, achieve sustained viral suppression, and mitigate the adverse effects of structural barriers to care such as lack of funds for transportation to the clinic (16–20). In addition, CCTs have been shown to help individuals expand their day-to-day financial choices, manage competing needs, and alleviate HIV care-related anxiety (20, 21). By removing the need for PWH to prioritize their money and time towards HIV-related care, CCTs may support feelings of autonomy and independence and give PWH increased decision-making options to that may allow them to optimize HIV care (20).

Although evidence points to the efficacy of incentives in improving healthy behaviors, little is known about what happens to PWH’s’ experiences with CCTs and their HIV care engagement when incentives are discontinued. The parent study, an Adaptive Strategy for Preventing and Treating Lapses of Retention in HIV Care (AdaPT-R, NCT#02338739), quantitatively examined how cessation impacts PWH and their continued engagement in care. The parent study findings suggest that the beneficial effects of incentives are time-limited, with cessation of CCTs resulting in deterioration in care engagement (22). Specifically, in AdaPT-R, results showed that starting CCTs at the time of ART initiation improved retention compared to standard-of-care (SOC) by 9.9% (absolute effect size; 95% confidence interval [CI]: 5.4%, 14.4%), but among those retained for a year, discontinuing CCTs led to 28.6% lower retention in care compared to continuing CCTs (absolute effect size; 95% CI:19.9%, 37.3%) (22). Further, a longitudinal individualized strategy of time-limited CCTs (continued until either initial lapse in retention or for a maximum of one year) did not improve treatment success compared to the care standard throughout (absolute effect size: −2.0%, 95% CI: − 8.2%, 4.2%).

This qualitative study aimed to elucidate PWH experiences and perceptions with CCTs for HIV care engagement, including effects after cessation, to investigate potential mechanisms of these results. Assessing PWH incentive experiences and perceptions with CCTs for care engagement will help us better understand incentive complexities and nuances and help determine the best use of CCTs as an intervention for sustained care retention.

## Methods

### Study population and setting

This qualitative study was nested within a larger parent trial, AdaPT-R (22). Study participants in the parent trial included adults (18 years and older) with HIV who had started ART within 90 days of study enrollment. The study took place in western Kenya (in Migori and Kisumu counties) in four government health facilities and one faith-based health facility. At study enrollment, participants were randomized to one of three stage 1 lower intensity interventions: standard of care (SOC), text message reminders (SMS), or conditional cash transfers (CCTs) in the amount of approximately $4 USD for each on time (−/+ 3 days) scheduled clinic visit. If participants in any of the stage 1 arms missed a clinic visit by 14 or more days during their first year in the study, they were randomized to a second time to a stage 2 higher intensity intervention. Those in the CCTs stage 1 intervention arm who did not miss a clinic visit by 14 or more days in their first year were considered successful participants who had not lapsed care. These individuals were then randomized again to either continue or discontinue CCTs and followed for one more year.

Participants were eligible for this sub-study if they were randomly assigned to receive CCTs at enrollment, did not lapse during their first year in the study, and were re-randomized to either stop or continue the CCT. We purposively selected participants using the maximum variation sampling approach to capture a broad and diverse range of perspectives from a subset of participants who had received CCTs for a year successfully. Sampling included a minimum of 10 participants for those who continued receiving and for those who discontinued receiving CCTs while factoring in age, gender, and health facility grouping for heterogeneity. Participants were approached and recruited for in-depth interviews during routine clinic visits within three months of randomization to continuation or discontinuation the CCTs.

### Data collection

Data collection took place at five health facilities in Migori and Kisumu counties from March 2017 through December 2018, within three months of participants’ randomization to continue or discontinue the CCTs. An experienced qualitative researcher who was a native speaker of local languages (Dholuo, Kiswahili, English) conducted the in-depth interviews using a semi-structured interview guide in the participants’ preferred language. The interviews were conducted in a private setting at the health facility to ensure privacy and confidentiality and took approximately 60–90 minutes to complete. Interview guide key topics, fully detailed in Additional File 1, included attitudes (e.g. reaction to incentive discontinuation on care engagement), experiences (e.g. influence of incentive on care attendance and engagement, challenges and barriers encountered in care engagement), and usage (e.g. how often the incentive was utilized for its intended purpose of mitigating clinic transportation costs). Interviews were audio-recorded, transcribed, and translated into English by the qualitative researcher. Quantitative data were extracted from the parent study database, including patient age, sex, education, marital status, distance to clinic, mode of transportation to the clinic, socio-economic factors, and if there was a lapse in care in the year following randomization to incentive continue or discontinue.

### Data analysis

Two experienced qualitative researchers coded the interview transcripts in Dedoose software (cloud based qualitative and quantitative analysis software developed by the Sociocultural Research Consultants (SCRC), Los Angeles, CA) with a codebook developed deductively based on the guide and refined inductively based on emerging codes identified in the initial transcripts. To ensure for inter-coder reliability and consistency, the researchers cross-coded the first three transcripts and met to review and resolve differences. Each researcher then coded independently and met weekly to compare codes and discuss and resolve discrepancies. After coding completion, excerpts were generated and analyzed inductively to identify theme saturation, with review and input from the principal investigators. Identified themes pertain to both study arms, with the exception of themes around the effects of CCTs discontinuation, which focus on the discontinued incentive arm only. Descriptive data containing participants’ sex, age, marital status, education level, walking time to clinic, mode of transport to clinic, socioeconomics, and lapses in care were extracted and summarized in Excel. Descriptive characteristics were generated to describe the populations within the CCTs continue and CCTs discontinue arms and collectively. Among those discontinued from receiving CCTs, lapse outcomes one year following randomization to stop the CCTs were compared to patient-reported perceived plans for future care in the qualitative data to examine if plans aligned with actual future care attendance in the year following their randomization. Illustrative quotes were identified for each emerging theme. Each quote excerpt includes information about patient age, sex, and study arm and whether the patient lapsed care (missed clinic visit by 14 days are more) in the year following randomization to either continue or discontinue the incentive.

### Ethics approval

The study was approved by the institutional review boards at the Kenya Medical Research Institute (KEMRI SSC No 2838) and the University of California, San Francisco (UCSF IRB No. 13–12810). All participants provided written informed consent.

## Results

This study included a subset of 38 participants who had received CCTs for one year from among the 517 successful non-lapsed participants. Of the 38 participants, 15 (39%) were randomized to continue CCTs and 23 (61%) were randomized to discontinue CCTs (Table 1). Half were female (N = 19) and median age was 30 years (range: 19–48); 29 were married or living with a partner and all 38 had at least some primary education. Taking a motorbike or walking were the most common ways sub-study participants travelled to the clinic; 32 required less than hour to walk to the clinic. Eight sub-study participants had electricity and none had running water in their homes. When exploring who lapsed in the year after randomization, 7 of 23 participants in the CCTs discontinue arm lapsed compared to 2 of 15 in the CCTs continue arm (Table 1).

Key themes from qualitative analysis are elucidated and presented below including care motivation, CCTs and decision-making, effects following cessation of the CCTs among the discontinue arm only, and participant recommendations on incentives.

### Care Motivation

We identified high intrinsic motivation to attend clinic visits among participants. Motivation to obtain and take their HIV medication, sustain good health, and survive for themselves and their families were many participants’ primary reasons for attending care. Most participants indicated they would come to the clinic regardless of the CCTs. Participants who missed clinic visits also expressed a strong motivation for future care attendance.

Broadly, participants conveyed how much they value their lives above all else and would not miss a clinic appointment to obtain their HIV medication, regardless of the CCTs.

*“HIV medication is my life, that is why I have never missed [a visit].”* Female, 31, CCTs Discontinued, Lapsed Care

*“I feel that nothing can stop me from coming to the clinic because I’m the one taking medication and I know how important it has been to me and so there is nothing that can prevent me from coming to the clinic.”* Female, 22, CCTs Discontinued, Lapsed Care

*“Even if the voucher will not be available, I will always make sure that I don’t miss my clinic [visit], this is because my life and the life of my children depend on my medication.”* Male, 40, CCTs Continued, Did Not Lapse Care.

Participants discussed how the CCTs were encouraging, but that care attendance was not hinged on their presence:

*“[The CCTs] encouraged me, yes, but at the same time, my coming was not pegged on it; at time I wouldn’t get the [CCTs] but I would still come and take medication without fail.”* Female, 43, CCTs Discontinued, Did Not Lapse Care

*“Coming for medication was my major reason for coming to the clinic; I cannot say that I was coming because of the [CCTs]; it motivated me, yes, but it wasn’t mandatory; I have still been able to come to the clinic.”* Male, 38, CCTs Discontinued, Lapsed Care

*“It [CCTs] motivated me to continue coming; however, I didn’t consider the transport alone but also my medications because my life depends on them. My number one priority is to get my medication.”* Female, 22, CCTs Discontinued, Lapsed Care

### CCTs and Decision-Making

Participants described ways the CCTs expanded their options and autonomy for decision-making for clinic attendance along with other daily needs. Although participants prioritized their clinic visits and health, it was not always easy given resource and work constraints. The CCTs allowed participants to entertain other possible ways to overcome resource and work constraints. The CCTs provided an added benefit by alleviating the transport burden to attend the clinic. Participants no longer had to struggle to find transport money to get to and from the clinic. Ultimately, knowing they would receive the CCTs gave participants more choices, reduced stress, and made care attendance easier. It also reduced reliance on others, particularly among those who lived too far to walk and with limited financial resources. Some participants also indicated that the CCTs brought financial relief by making up for pay lost at work and provided a little extra money to buy food and other necessities. Although the CCTs provided extrinsic motivation by increasing their decision-making options and making clinic attendance easier, it appeared to supplement intrinsic care motivation for most participants.

One participant discussed her challenges with clinic transportation and the ways that the CCTs helped alleviate this burden:

*“Because I wouldn’t have suffered looking for transport to and from the clinic like I sometimes do; I can borrow transport from someone and repay them once I am from the clinic but it’s a challenge, it’s just that my desire for HIV medication overrides any prevailing challenge. I must work a way out to get to the clinic.”* Female, 22, CCTs Discontinued, Lapsed Care

Another participant discussed how the incentive helped him in clinic transportation:

*“[The CCTs] ease my burden to some extent that would make it easier for me to come and go back home. That is the way it helped me mostly.”* Male, 23, CCTs Discontinued, Lapsed Care

For participants who faced physical challenges reaching the clinic, the transportation incentive was especially helpful:

*“It helped me with covering up my transport cost to the hospital since I come from far and I’m not able to walk; I don’t have a proper job for now and so I always have to ensure that I have a means of getting money to reach the hospital; the [CCTs] has really been helpful.”* Male, 41, CCTs Continued, Did Not Lapse Care

Aside from covering the intended purpose of transport costs, many other participants discussed how leftover money from the CCTs allowed them to purchase food and other household necessities:

*“[The CCTs] encouraged me because at times I could even lack salt in my house so when my clinic day comes I would say to myself to go to the clinic, I will find money there.”* Female, 29, Migori, CCTs Discontinued, Lapsed Care

*“It motivated me because I would spend the money for the benefit of my house; as you know, money is the pillar of happiness in a family.”* Male, 27, CCTs Continued, Did Not Lapse Care

One participant described how taking food with his HIV medications reduced his side effects and that he used leftover money from CCTs to buy that food:

*“[The CCTs] helped so much; I could use part of the money to buy a quarter of meat and change my diet; there drugs require someone to have a good diet.”* Male, 48, CCTs Discontinued, Did Not Lapse Care

The leftover incentive money also helped participants fulfill familial responsibilities, as described by one mother:

*I was given Ksh. 400(~$4) and two way I used Ksh. 100 (~$1) the balance of Ksh. 300 (~$3) I would use for buying sugar for my children, I would use it to buy food, silver cyprinid, sometimes I would buy cooking oil.”* Female, 29, CCTs Discontinued, Lapsed Care

Other participants mentioned how the CCTs helped make up for money earned at work or by offsetting missed income.

*“It influenced my decision to come to the clinic because I couldn’t have an excuse for missing to come; I was certain of getting some money at the end of the day even if I didn’t open business that day.”* Female, 31, CCTs Discontinued, Did Not Lapse Care

*“I felt happy because whenever I missed making profit in my business due to the appointment the voucher I received would cater for that loss.”* Female, 31, CCTs Discontinue, Lapsed Care

### Clinic attendance upon CCTs discontinuation

The parent AdaPT-R study to this sub-study demonstrated that CCTs were effective at supporting care engagement while they were provided, yet following their discontinuation, engagement worsened (22). This sub-study’s findings revealed that despite high intrinsic motivation to attend clinic visits regardless of the CCTs, cessation appeared to increase the burden of accessing care for some participants, particularly among those with constrained financial situations and/or living far way. The incentives had been instrumental in making care access easier by relieving structural burdens. Once the incentive stopped, participants had fewer options. They had to revert to relying on others by borrowing transport money, walking long distances to the clinic, and in some cases miss clinic visits. Many participants expressed disappointment, sadness and increased stress when the CCTs ended.

The cessation of the CCTs directly led to missing a clinic visit due to the inability pay for transportation to the clinic, as illustrated by this participant:

*“Like this past month, I did not visit because I did not have transport. So, it would help me and I would go on time and not be late. This past one I missed because I did not have enough transport... there is nothing that can hinder me from coming to the clinic except when I am not able to raise the fare because this place is far.”* Female, 27, CCTs Discontinue, Did Not Lapse Care

Disappointment and stress upon the cessation of CCTs were common reactions; participants were sad and troubled by having a new or re-emerging burden of finding money or a way to get to clinic appointments, including relying on other family members.

*“Some part of my heart sank...no job, hard to find money to get to clinic; have to rely on mum and husband; but will continue with care.”* Female, 21, CCTs Discontinued, Did Not Lapse Care

*“It was a challenge because I had to strategize on how I would come to the clinic; sometimes I just don’t have money to use for transport to come to the clinic; while I was still receiving the [CCTs], I could sometimes borrow money from someone with the surety that I would return the money once I came back from the clinic.”* Male, 46, CCTs Discontinued, Did Not Lapse Care

Additionally, for some participants the unexpected CCTs cessation caused them to be stranded near the clinic as they looked for funds to get home:

*“I didn’t feel well because I had expectations. That’s the money I had planned to use as transport to get back home; I didn’t go back to Siaya on that day and so I spent three days at my friend’s house as I was looking for my means of transport back home.”* Male 27, CCTs Discontinued, Did Not Lapse Care

Participants spoke of the ease CCTs afforded them— accessing care was no longer a problem—yet their struggle for transportation re-surfaced after the CCTs stopped, particularly among those coming from far.

*“It [CCTs] eased my burden to some extent that would make it easier for me to come and go back home. That is the way it helped me mostly… I would like for it to be reinstated because it made it easier for me to move about”* Male, 23, Rongo, CCTs Discontinue, Lapsed Care

One participant also noted a concern about perceived stigma following CCTs cessation— without the incentive and the autonomy it provided, other community members may now learn about her HIV status as she turns to them to borrow money for clinic transport.

*“Sometimes I have to borrow money for transport to come to the clinic, somebody might wonder why I come to the clinic regularly and might join the dots and get to know that I am living with HIV.”* Female, 22, CCTs Discontinued, Lapsed Care

### CCTs recommendations by participants

When asked about incentives in the future, some participants advised against its deployment while others wished it could be resumed. There was a feeling that if you live far and are poor and used the funds for transport, one could easily default from their appointments, eventually become ill or die, and blame the hospital for not meeting their transportation needs if the CCTs were not resumed. Others expressed concern that the CCTs could foster dependency. The start and stop of the CCTs also sent a confusing message; participants recognized it as being there to motivate clinic attendance, yet it was then taken away. Those wishing the CCTs could be resumed felt it would continue to motivate care attendance, alleviate transportation challenges, and also help with lost wages and food.

*“The truth is that people are very poor and being given transport at the facility and ease the burden that comes with living with HIV but it can create dependence.”* Female, 22, CCTs Discontinued, Lapsed Care

*“You do issue us transport cash which I thought was to motivate us to come to the clinic but later you stopped it. It is generally too complicated to be understood”* Female, 31, CCTs Discontinued, Lapsed Care

*“I would like for it to be reinstated because it made it easier for me to move about”* Male, 23, CCTs Discontinued, Lapsed Care

## Discussion

This study helps elucidate why patients’ care engagement may be compromised after a financial incentive is discontinued, as observed in the parent AdapPT-R study. This sub-study found that patients were highly motivated and had the best intentions to attend clinic visits, yet motivation and intent were not enough when hardship in accessing care resurfaced after cessation of CCTs. As behavioral economics has shown, individuals tend to make decisions based on practicalities and current circumstances, not necessarily on what is best for their well-being and future health (23). If resources are scarce, individuals have fewer choices and may be not be able to prioritize their health or consider the risks involved in their decisions (24). Unaddressed barriers to care, such as insufficient resources for transportation, may interfere with clinic attendance (25–27). A qualitative study in Uganda illustrated the unending challenge of finding funds to travel to the clinic, resulting in difficult life choices (25). A study carried out in Mexico found that cash incentives were effective when in place, but once stopped, engagement in HIV care dropped significantly (28). The CCTs in this study were a structural intervention that expanded patients’ options and autonomy to exercise choice in decision-making while it was provided (20, 24). The benefit of more choice options and less worry about finances was expressed by participants who felt empowered by the CCTs; it allowed them to prioritize their HIV clinic attendance with more ease. Discontinuing the CCTs contributed to feelings of defeat when patients experienced a sudden decline in their decision-making options, leaving them unable to prioritize their HIV care (24).

We anticipated that the CCTs would nudge patients toward healthy behaviors, shifting patients from extrinsic to intrinsic motivations for care continuity (23). However, we found little evidence supporting this hypothesis. CCTs have been shown to prompt effective increases in HIV testing and linkage to care behaviors, the first steps in the care cascade (29–34). In our study, futher along the care cascade, it was difficult for patients to shift towards better clinic attendance continuity when the structural barriers that time-limited CCTs mitigated, resurfaced. In the face of persistent structural barriers, evidence suggests CCTs may not be enough to ensure sustained behavior change (35). Interventions that address the structural barriers with long-term change will more readily support sustained clinic attendance for PWH. As the behavioral economist Thaler has advised “If you want to people to do something, make it easy”(26). It is essential to determine what is standing in the way of a desired behavior and then clear the path (26, 27).

Giving an incentive, then taking it away, may make it more difficult for people to do what they want (27). Renewed hardship in accessing care may generate unintended harm. Rather than place PWH back in neutral where they started, the cessation of CCTs may have set them back further by fostering unsustainable economic dependency on the incentive. Pre-incentive, PWH may have coped with the resources at hand, then got used to the new norm of additional resources and options with the CCTs, only to have it removed without a mechanism to address their structure barriers. This may explain, in part, why participants in the AdaPT-R parent study had worse engagement than those in standard of care after the CCTs were removed (22). Without a plan or resources for transportation after its removal, participants may have been faced with making difficult choices between spending resources on life necessities or transportation to the clinic. The temporary administration of CCTs gives PWH a sense of what it’s like to be more financially secure, and then takes that autonomy away.

These results have implications regarding the sustainability and recommended usage of CCTs. Though CCTs may help some PWH form good clinic attendance habits while they are consistently being provided, once removed that benefit diminishes. The financial burden PWH in SSA face are pervasive, and without an actively method of addressing these burdens (i.e. CCTs), motivation alone is not enough to remain in care. Moreover, CCTs may in fact cause additional harm if they are only given temporarily. CCTs may be most beneficial for PWH if they are given consistently to provide reliable help to address their financial constraints to getting to and from the clinic. Due to the high monetary costs of giving CCTs long-term, a targeted approach that assesses which PWH would benefit the most from CCTs may be more viable. For instance, a gradual incentive phase out approach, where the CCTs are decreased as care constraint reduction is demonstrated, could be considered (16). Other options such as home or community delivery of ART could alleviate structural issues related to care engagement without the challenge of incentive durability. One economic evaluation of home delivery of ART in Zimbabwe suggested that for ART-stable PWH, community-based models cost less for both providers and PWH and did not impact care outcomes (36). Other studies in sub-Saharan Africa have similarly suggested that community-based ART models provide comparable care outcomes when compared to traditional facility care and may be cost-neutral or cost-effective (37–39). It is possible that these community-delivery methods may be more cost-effective than paying for second- and third-line treatments that are necessary for patients who have missed critical appointments.

### Limitations

Only PWH who were already attending care were included in the study; thus, the PWH who may have been suffering the most from barriers to care attendance, and may have benefited most from the CCTs, were not included. As with many qualitative, interview-based studies, there is always the risk of social-desirability bias influencing participants’ reports about their motivation.

## Conclusions

This study helps us better understand the durability of financial incentives in the context of HIV care engagement, particularly when incentives end. Participants in this study prioritized their health and were intrinsically motivated to remain in care even if the incentive ceased, yet once the time-limited incentives were removed, hardships to accessing care re-emerged for some participants, particularly for those with unaddressed structural barriers. Together with the quantitative findings in the parent AdaPT-R study, these results support the idea that careful consideration be exercised when implementing incentives for sustainable engagement effects.

## Figures and Tables

**Table 1:** Participant Characteristics

Characteristics	CCTs continue N = 15	CCTs discontinue N = 23	Total N = 38
**Sex**			
Females	7	12	19
Males	8	11	19
**Age**			
Median age	33	30	30
Range	21–47	19–48	19–48
**Marital status**			
Married/living together	14	15	29
Single/separated/divorced	1	6	7
Widowed	0	2	2
**Education level**			
Some primary	11	10	21
Some secondary	3	12	15
Some college	1	1	2
**Time to clinic in walking minutes**			
60 minutes or more to walk to clinic	0	6	6
60 minutes or less to walk to clinic	15	17	32
**Mode of transport**			
Walking	5	11	16
Bodaboda/pikipiki (motorcycle)	8	11	19
Matatu (minibus)	2	0	2
Tuk-tuk	0	1	1
**Socio-economics**			
Electricity in the home	3	6	8
Running water in house	0	0	0
Food insecure mean score (range) (0 = low & 18 = high)	6.00 (0–12)	9.57 (2–17)	8.16 (0–17)
**Lapsed care in the year following CCTs randomization to continue or stop**			
Did not lapse	11	15	26
Lapsed care	2	7	9
Missing	2	1	3

Characteristics of participants in this qualitative study who received CCTs for one year and did not lapse care and were then randomized to continue with the CCTs or discontinue the CCTs and followed for one more year. (Table 1)

## Data Availability

The datasets used and/or analysed during the current study are available from the corresponding author on reasonable request.

## Abbreviations

HIV: Human Immunodeficiency Virus
AdaPT-R: Adaptive Strategy for Preventing and Treating Lapses of Retention in HIV Care
CCT: Conditional Cash Transfer
ART: Antiretroviral Therapy
PWH: People with HIV
SSA: Sub-Saharan Africa
SMART: Sequential Multiple Assignment Randomized Trial
RCT: Randomized Control Trial
SOC: Standard of Care
ATE: Average Treatment Effect
CI: Confidence Interval
SMS: Short Messaging Service/ Text Message Reminders
USD: United States Dollars
SCRC: Sociocultural Research Consultants
Ksh: Kenyan Shilling
